# Aeroponics enables consistent cutting propagation of kratom (*Mitragyna speciosa*): impacts of photoperiod, cultivar, and rooting hormone

**DOI:** 10.3389/fpls.2025.1650327

**Published:** 2025-08-29

**Authors:** Mengzi Zhang, Cynthia Montanez, Brian J. Pearson, Yuncong Li, Jianjun Chen

**Affiliations:** ^1^ Mid-Florida Research and Education Center, Environmental Horticulture Department, University of Florida, Apopka, FL, United States; ^2^ Mid-Columbia Agricultural Research and Extension Center, College of Agricultural Sciences, Oregon State University, Hood River, OR, United States; ^3^ Tropical Research and Education Center, Department of Soil, Water, and Ecosystem Sciences, University of Florida, Homestead, FL, United States

**Keywords:** controlled environment, genotype, hydroponic, medicinal crops, root morphology, root scanning

## Abstract

**Introduction:**

Kratom (*Mitragyna speciosa*) has gained increasing attention for its potential to manage pain and alleviate opioid withdrawal symptoms. Despite growing interest, production practices, including vegetative propagation, remain underexplored. This study compared indoor aeroponics with greenhouse mist systems for rooting kratom cuttings and evaluated the effects of photoperiod, rooting hormone, and cultivar on the rooting success.

**Methods:**

In Study I, stem cuttings collected from ‘MR-Malaysian’ over two seasons, 180 each, were rooted in aeroponic units, 15 cuttings per unit. Twelve aeroponic units were randomly assigned to 10-, 14-, or 24-hour photoperiods in environmentally controlled growth rooms, with four units per room. In parallel, cuttings from the same cultivar were evaluated in a greenhouse mist system across three seasons. In each season, 60 cuttings were rooted in four trays, 15 each. In Study II, cuttings derived from three cultivars (MR-Malaysian, DR-Bumblebee, and Hawaii) were tested simultaneously using the indoor aeroponic and greenhouse mist systems, with or without rooting hormone treatments (5 mg/L IBA and 2.5 mg/L NAA). A series of rooting parameters was collected during the two studies.

**Results:**

A 14-hour photoperiod significantly enhanced root initiation and root growth compared to a 10-hour photoperiod, as indicated by increased root length, area, branching, and biomass. The aeroponic system consistently promoted high rooting percentages (85%-92%) and root growth, whereas the greenhouse mist system exhibited high seasonal variability (7%-98%) and inconsistent rooting success. In Study II, ‘MR-Malaysian’ overall outperformed the others, exhibiting high rooting rates and root growth with a relatively short production cycle, as well as being less susceptible to *Fusarium*. Hormone application increased the number of roots, but it had minimal effects on other parameters.

**Discussion:**

Our results showed that the aeroponic system consistently yielded a significantly higher rooting percentage and better rooting quality compared to the greenhouse mist system across the three cultivars. Such qualities included greater final root number, root dry mass, root length, root area, root volume, and higher numbers of root tips, forks, and crossings. However, the aeroponic system may pose a possibility of spreading pathogens. This study, for the first time, demonstrates that photoperiod, rooting system, and cultivar are crucial factors in rooting kratom cuttings. The aeroponic system represents a new and effective way of propagating kratom cuttings year-round.

## 1 Introduction

Kratom (*Mitragyna speciosa*) is a tropical evergreen tree in the Rubiaceae family, native to the humid wetland forests of Southeast Asia including southern Thailand, Malaysia, Indonesia (notably Borneo and Sumatra), and parts of Myanmar. It has also been introduced to other regions such as the Philippines and New Guinea (Takayama, 2004; Eisenman, 2014; Cinosi et al., 2015). The leaves of kratom are rich in bioactive alkaloids and have been used by outdoor laborers in Southeast Asia as a stimulant and mood enhancer, helping them endure long hours of physical labor while under intense climatic conditions (Singh et al., 2019). Beyond its stimulant properties, kratom has also been used to treat various ailments, including pain, diarrhea, hypertension, fever, and wounds, as well as to alleviate opioid withdrawal symptoms (Cinosi et al., 2015; Singh et al., 2017, 2019).

During the last two decades, kratom has gained increasing attention in Western countries. Historically, Southeastern Asians have utilized the analgesic properties of fresh leaves by chewing on or steeping them in hot water to brew tea [Eisenman (2014); Huisman et al., 2023]. Over time, concentrated kratom leaf extracts have been produced as an oral supplement to alleviate pain and sold to countries that do not have kratom naturally (Brown et al., 2017; Sharma et al., 2019; Huisman et al., 2023). The rise in kratom use in the West is largely linked to the ongoing opioid crisis, with many individuals turning to kratom for relief of pain, anxiety, and depression (Garcia-Romeu et al., 2020). Similar to its traditional uses, some Western users also rely on kratom to reduce opioid consumption (Garcia-Romeu et al., 2020). Approximately 10–15 million people in the U.S. are estimated to consume kratom regularly (Henningfield et al., 2022), which requires the importation of nearly 2,000 metric tons each month (Shah et al., 2021). As a result, the U.S. kratom industry generates $1.2 to $5.0 billion in revenue annually (Botanical Education Alliance and American Kratom Association, 2016). However, there is no commercial production of kratom in the U.S., and no cultivation protocol is available for kratom propagation and production.

Propagation is a crucial initial step in the crop production cycle. Due to low seed viability and poor germination rates, kratom is propagated through stem cuttings. The success in cutting propagation is influenced by several factors, including plant genetics (Leakey et al., 1994; Marques et al., 1999), mother stock plant health and age (Tchoundjeu et al., 2002; Bhardwaj and Mishra, 2005; Amri et al., 2010), temperature (Owen and Lopez, 2018), humidity and vapor pressure deficit (Will et al., 2013), light (Craver et al., 2019; Owen and Lopez, 2018), season (Ayoub and Qrunfleh, 2006), cutting positions (Bhardwaj and Mishra, 2005), hormone application (Tchoundjeu et al., 2002; Bhardwaj and Mishra, 2005; Amri et al., 2010), rooting substrate composition (Tchoundjeu et al., 2002), and propagation systems (Tokunaga et al., 2020; Weingarten et al., 2024). To our knowledge, no studies to date have explored the factors influencing kratom’s vegetative propagation.

Rooting hormones and rooting substrates can significantly affect rooting success. Applications of synthetic rooting hormones, such as indole-3-butyric acid (IBA) and naphthalene acetic acid (NAA), to stem cuttings generally accelerate initial root development, improve rooting uniformity, and reduce rooting times by mimicking their natural auxin, indole acetic acid (IAA) (Cerveny and Gibson, 2005; Owen and Lopez, 2018). Thus, IBA and NAA have been used to propagate a wide range of crops, including coleus (*Coleus scutellarioides*), camellia (*Camellia japonica*), euonymus (*Euonymus kiautschovicus*), chrysanthemum (*Chrysanthemum × morifolium*), pfitzer juniper (*Juniperus × pfitzeriana*), Manetti rose (*Rosa × noisetteana*), and apple (*Malus domestica*) (Blythe et al., 2007). Soilless substrates formulated by optimizing peat, vermiculite, and perlite in different proportions are used for rooting stem cuttings. A higher percentage of peat in the substrate significantly increased root number and the length of the longest root in *Ilex ×meserveae* (Maynard, 2000) and improved rooting percentage, root length, and root quality score in *Lobostemon fruticosus* (Swarts et al., 2018). Vermiculite, which offers both good water retention and aeration, has been shown to improve rooting of tropical crops such as spiked pepper (*Piper aduncum*) (Gomes and Krinski, 2018), oleander (*Nerium oleander*) (Ochoa et al., 2002), and papaya (*Carica papaya*) (Kaity et al., 2007). In our preliminary trials, different concentrations of rooting hormones and substrate compositions (combinations of peat, perlite, and vermiculite) were evaluated for rooting kratom cuttings. Results indicated that substrate composition had minimal effect on rooting success and cutting quality. Higher hormone concentrations (e.g., 2,500 mg/L IBA/1,250 mg/L NAA and 7,500 mg/L IBA/3,750 mg/L NAA) provided limited or even negative effects on rooting, suggesting that further investigation into lower hormone concentrations was warranted.

Light plays a vital role in root initiation and subsequent plant growth. Higher photosynthetic daily light integral (DLI), achieved through longer photoperiods or supplemental lighting, can significantly enhance callus formation and rooting quality. Currey et al. (2012) found that an increase of DLI from 1.2 to 12.3 mol·m^-2^·d^-1^ led to a substantial increase in root dry mass by 156% to 1137% across nine tested species and improved the quality index by 176% to 858%. Similarly, an additional DLI of 3.6 mol·m^-2^·d^-1^ supplemented by LED lighting significantly enhanced the rooting percentage, root length, root number, and root dry mass of carnation (*Dianthus caryophyllus*) cuttings in 10 days, and further increased root dry mass 15 days later (Wang et al., 2020). While a DLI of 3–8 mol·m^-^²·d^-^¹ is generally recommended for callusing and 5–10 mol·m^-^²·d^-^¹ for root development, these recommendations are primarily based on studies involving floriculture crops (Faust et al., 2016). More recent research suggests that a DLI of 10 to 12 mol·m^-^²·d^-^¹ is optimal for cutting propagation of culinary herbs (Kohler and Lopez, 2021). While much of the existing research on light requirements during propagation has focused on increasing DLI through supplemental lighting, relatively little attention has been given to the role of photoperiod. Preliminary observations from our team suggest that kratom may be photoperiod sensitive. Therefore, understanding the impact of photoperiod on kratom propagation is essential for developing effective lighting guidelines.

**Figure 1:**
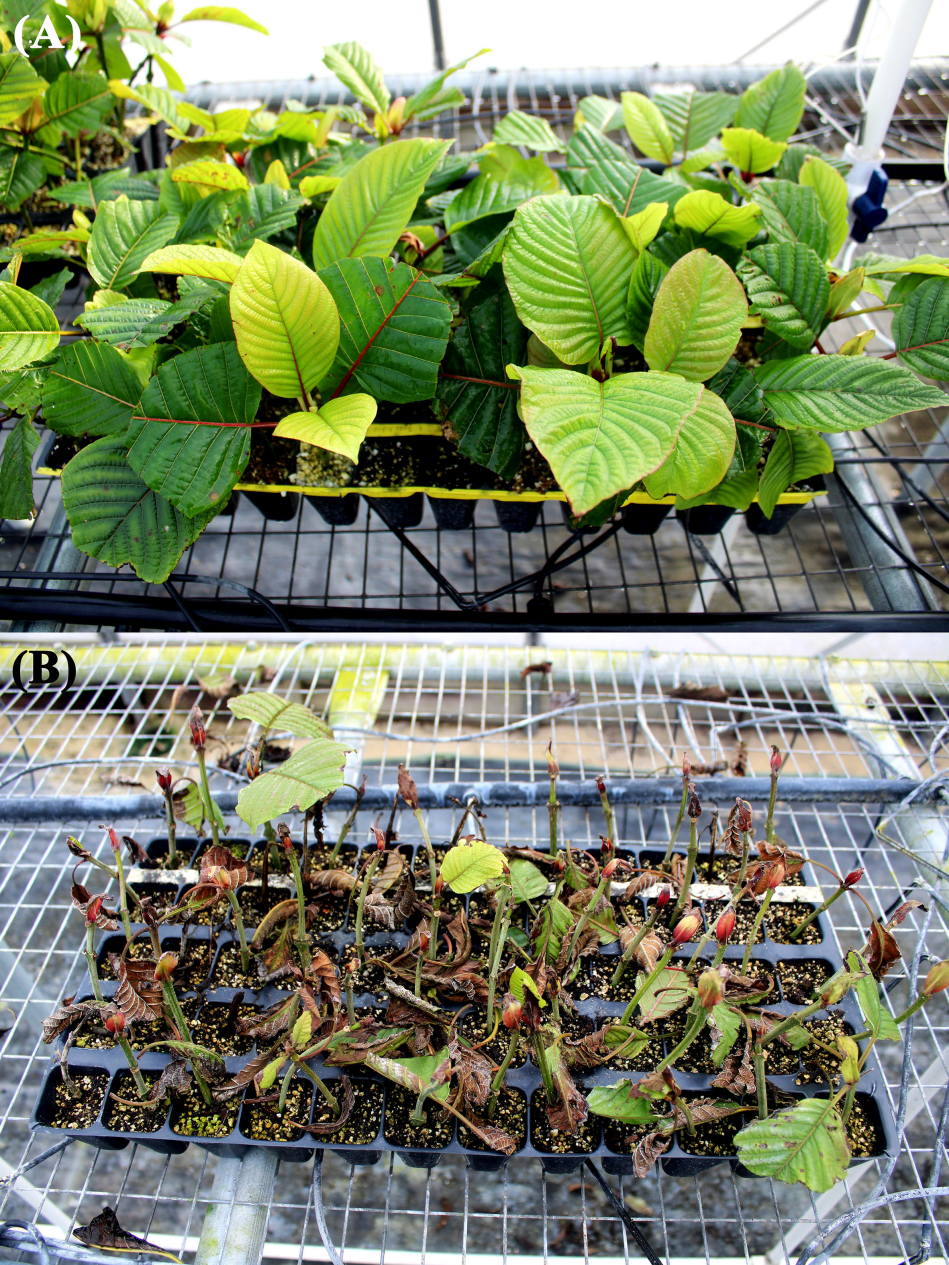
Example of a *Mitragyna speciosa* cutting propagation trial under the mist in a greenhouse. The initial cutting condition **(A)** and 8 weeks after rooting **(B)**.

Overhead misting is a widely adopted method in greenhouse propagation of cuttings. This approach often involves intermittent misting on benches to maintain humidity around cuttings. However, excessive misting can cause waterlogging and oxygen deficiency, resulting in poor rooting efficiency. The use of overly cold water for misting can lower soil temperature and slow down rooting. Multiple preliminary trials conducted by our research team using a greenhouse misting method yielded suboptimal results (**Figure 1**), marked by low rooting success, high variability, and prolonged rooting process (8–10 weeks). These outcomes highlight the need to explore alternative propagation systems for more consistent and efficient propagating kratom. Recently, new techniques, such as aeroponics, have been developed for improving rooting efficiency. Aeroponic technology offers several advantages, including superior aeration in the root zone, enhanced water and nutrient use efficiency, year-round cultivation, and a faster production cycle (Kumari and Kumar, 2019). Mehandru et al. (2014) demonstrated that an aeroponic system without the use of IBA significantly improved the rooting of *Leptadenia reticulata*. The rooting percentage increased by 38%, the number of roots per cutting by 2.9 times, and the length of roots per cutting by 1.7 times compared to conventional soilless propagation media. Similarly, Weingarten et al. (2024) reported that aeroponic propagation produced significantly better root quality in two *Cannabis sativa* cultivars compared to foam and rockwool media. Additionally, aeroponic propagation is typically conducted indoors, where environmental conditions are more stable and easily controlled. This indoor setting offers additional benefits, such as reducing crop losses, increasing productivity per area, and accelerating rooting (Gibson et al., 2020). These advantages make aeroponic systems an appealing option for propagation of kratom cuttings.

With the rising interest and demand for kratom, coupled with a lack of foundational knowledge on its propagation, the objectives of this study were to (1) compare the rooting success of an indoor aeroponic system with a greenhouse mist system; (2) examine the effects of different photoperiods on root development; (3) investigate the impact of rooting hormone on propagation of three cultivars in both systems; and (4) assess rooting success and performance among three kratom cultivars.

## 2 Materials and methods

### 2.1 Study I – photoperiod and seasonal trial

#### 2.1.1 Plant materials

The stock plants of ‘Mitragynine-Rich (MR)-Malaysian’ were initially propagated from seed. At the time of this experiment, they were three years old and grown in 57-L containers filled with a soilless substrate composed of 25% Florida peat, 25% Canadian peat, 30% pine bark, and 20% perlite based on volume (Reliable Peat Company, Leesburg, FL, USA). Plants were cultivated under a shade structure providing a 75% reduction in ambient sunlight (25% full sun) in Apopka, Florida (latitude 28°38′ N, longitude 81°33′ W). They were fertilized every six months with 200 grams of Osmocote 15-9-12, an 8–9-month controlled-release fertilizer (Scotts, Marysville, OH, USA). Before taking cuttings, plants were drenched weekly for three weeks with Peters Professional 20-20–20 soluble fertilizer (ICL, Everris NA Inc., Dublin, OH, USA) at a nitrogen concentration of approximately 357 mg/L. They were watered daily through drip irrigation, supplemented occasionally by rainfall.

A total of 240 tip cuttings were collected from three healthy, pest-free stock plants. The selection of the plants was based on specific criteria: green, healthy foliage; vigorous, actively growing tips; and no flower buds or flowers. Only semi-hard tips (firm but not fully lignified) were chosen to ensure optimal physiological status for vegetative propagation. Cuttings were taken in the morning before 10 a.m. under shade conditions to minimize water loss. Each cutting was approximately 15 cm long and taken from the first two nodes below the apical meristem. To ensure pest-free, the cuttings were initially submerged in soapy water for approximately one minute, followed by rinsing in clean water. Each cutting was then given a 45-degree cut to enhance vascular tissue exposure, and the leaves at the apical node were trimmed in half to reduce transpiration. Immediately after cuttings were made, they were manually sprayed with water on both the leaf surfaces and bases to reduce water stress before placement under experimental treatments. Cuttings from each stock plant were evenly distributed to each treatment to minimize potential confounding effects of genetic variation among source plants.

#### 2.1.2 Treatment and experiment design

**Figure 2:**
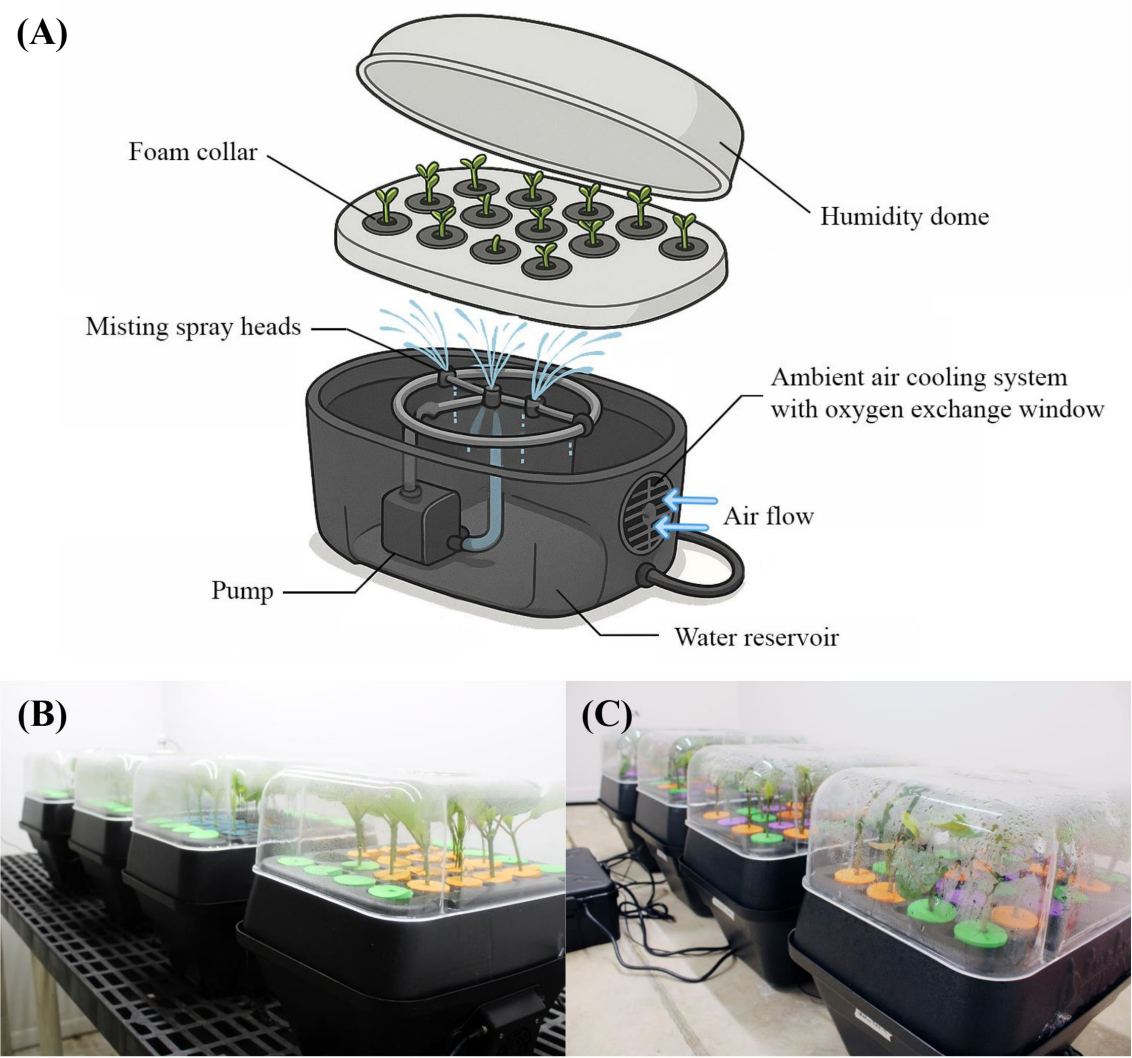
Aeroponic units used in the rooting of *Mitragyna speciosa* stem cuttings. An illustration of the aeroponic unit **(A)**. The setup of aeroponic units indoors for Study I **(B)** and Study II **(C)** experiments.

The experiment was carried out simultaneously in three identical indoor growth rooms and a shaded greenhouse mist bench. Three rooms were provided with three photoperiods: 10, 14, and 24 hours, respectively, resulting in corresponding DLIs of 4.4 ± 0.1, 6.3 ± 0.1, and 10.5 ± 0.2 mol·m^-2^·d^-1^. There were four T-24 aeroponic units (TurboKlone System, Sparks, NV, USA) in each room, and 15 cuttings were placed upright in polyethylene collars per T-24 aeroponic unit (**Figure 2**). Each unit contained an equal number of cuttings taken from the same stock plants to minimize genetic variability. Each aeroponic unit was equipped with a pump to deliver continuous misting at the base of the cuttings. Closely fitting humidity domes were used to maintain high humidity levels and minimize water loss from the leaves. In parallel, 60 cuttings were rooted in four trays, with 15 cuttings per tray, derived from three stock plants. The trays were placed on a misting bench of a shaded greenhouse. A black shade net with 70% light exclusion was installed within the greenhouse. A soilless substrate consisting of 80% vermiculite and 20% perlite (v:v) was used, which was selected based on findings from preliminary trials. An additional sacrifice tray containing 18 cuttings was used for weekly photographic documentation, with three cuttings removed from the tray and checked for rooting each week over six weeks.

The study was conducted over three seasons, with Season 1 initiated in May 2024 and Season 2 in October 2024. The third season was conducted only in the greenhouse, beginning in February 2025. The experiments for both the indoor aeroponic and greenhouse mist systems used a completely randomized design. In the indoor aeroponic system, each season served as a replication, consisting of three photoperiod treatments (three rooms). In the greenhouse mist system, four trays were used as replications within each season.

#### 2.1.3 Environmental conditions

In the indoor growth rooms, the photoperiod was provided by white full-spectrum LEDs (VYPR 2p; Fluence Bioengineering, Inc., Austin, TX, USA) regulated by timers (BN-LINK Inc., Cucamonga, CA, USA). Photosynthetic photon flux density (PPFD) was measured with a quantum sensor (MQ-500; Apogee Instruments Inc., Logan, UT, USA) at six representative positions within each aeroponic unit at the cutting canopy level. The measurement was carried out without the humidity dome due to equipment constraints. However, the dome is translucent, and its effect on PPFD is minimal, less than 5 µmol·m^-^²·s^-^¹. The average PPFD the cuttings received under 10-, 14-, and 24-hour treatment was 121 ± 1.8, 125 ± 2.1, and 122 ± 2.5 µmol·m^-2^·s^-1^, respectively. Air temperatures were targeted at 25°C and maintained using air conditioners, with temperatures monitored by thermocouples and recorded every 10 minutes by a wireless data logging station (HOBO RX3000; Onset Computer Corporation, Bourne, MA, USA). High humidity was maintained using closely fitting humidity domes, and a fine water mist was manually applied several times daily. To verify optimal conditions, relative humidity inside the domes was monitored for 24 hours prior to the start of the experiment, without cuttings, using data loggers equipped with humidity sensors (GSP-6G, Elitech, San Jose, CA, USA; WatchDog 2475, Spectrum Technologies, Inc., Aurora, IL, USA). The average relative humidity ranged from 95% to 99%. Cuttings were inspected multiple times per day to ensure the presence of fine condensation on the inside of the domes, indicating consistently high humidity levels. Additionally, the pH and electrical conductivity (EC) of the water in each aeroponic unit reservoir were adjusted to 5.6-5.8 and 0.45-0.5 S/m, respectively, at the start of the experiment and were monitored weekly.

In the greenhouse, plug trays were randomly arranged on a mist bench and subjected to natural daylight and day length under a black shade cloth with 70% light exclusion. Misting was applied for five seconds every five minutes between 6 a.m. and 7 p.m. Air temperature and humidity were measured and recorded every 10 min while PPFD was recorded every 15 min using data loggers (GSP-6G, Elitech, San Jose, CA, USA; WatchDog 2475; Spectrum Technologies, Inc., Aurora, IL, USA). Average air temperature, instantaneous PPFD, DLI, and humidity levels for both the greenhouse and indoor environments are detailed in **Table 1**.

**Table 1:** Environmental conditions [average air temperature, photosynthetic photon flux density (PPFD), daily light integral (DLI), and relative humidity] across seasons and treatments for *Mitragyna speciosa* propagation in Study I and II.

Study	Location	Season	Treatment	Air temperature (°C)	PPFD (µmol·m^-2^·s^-1^)	DLI (mol/m^-2^·d^-1^)	Humidity (%)
I	Indoor	Season 1	10 hours	24.7 ± 0.03	121.0 ± 0.8	4.4 ± 0.1	NA
			14 hours	24.8 ± 0.03	125.2 ± 2.1	6.3 ± 0.1	
			24 hours	24.8 ± 0.03	122 ± 2.5	10.5 ± 0.2	
		Season 2	10 hours	24.3 ± 0.03	123.8 ± 3.6	4.5 ± 0.1	NA
			14 hours	24.3 ± 0.03	124.5 ± 3.0	6.3 ± 0.2	
			24 hours	25.6 ± 0.03	122.1 ± 2.7	10.5 ± 0.2	
	Greenhouse	Season 1	NA	25.7 ± 0.02	51.2 ± 0.9	4.4 ± 0.1	97.9 ± 0.06
		Season 2		25.4 ± 0.02	32.1 ± 0.6	2.8 ± 0.1	97.0 ± 0.08
		Season 3		18.8 ± 0.05	38.6 ± 0.7	3.3 ± 0.1	98.4 ± 0.05
II	Indoor	NA	Water	24.5 ± 0.02	121.5 ± 1.7	6.1 ± 0.1	NA
			hormone		122.8 ± 1.7	6.2 ± 0.1	
	Greenhouse		Water	20.1 ± 0.05	49.5 ± 0.9	4.3 ± 0.1	97.6 ± 0.07
			hormone				

NA, Not applicable. PPFD for the indoor environment was measured at the cutting canopy level without humidity domes. Humidity for the indoor environment was NA but is estimated to be between 95-99%.

#### 2.1.4 Data collection and analysis

The experiment lasted six weeks, during which the following data were collected. Days to root (d) were monitored daily in the indoor environments and recorded based on the first visible appearance of roots. Representative photos of rooting progress were taken weekly in both the greenhouse and indoor environments. At the end of the experiment, the rooting status of the cuttings was evaluated using a Boolean assessment to calculate the rooting percentage (%). The number of new leaves was counted, and leaf area (cm²) was measured using a leaf area meter (LI-3000; LICOR, Inc., Lincoln, NE, USA). Roots were carefully cut from the base of the cuttings. Various root metrics, including total root length (cm), projected area (cm^2^), surface area (cm²), average root diameter (mm), and root volume (cm³) were recorded for rooted cuttings collected from both the greenhouse and indoor environments using a root scanning machine (J221B Perfection V850 Pro Photo Scanner, Epson, Los Alamitos, CA, USA), and image was processed with WinRHIZO software (Regent Instruments Inc., Quebec, Canada). The number of tips, forks, and crossings was recorded only in the indoor environment, as image-based predictions for these parameters in the greenhouse were not sufficiently accurate. Root dry mass (g) was measured with a lab scale (PL601-S, Mettler Toledo, Switzerland) after drying in the oven (Model 40 GC, Burr Ridge, IL, USA) at 80 °C for 5 days.

For gathering aeroponic data, the above parameters were recorded individually for each cutting, and the means of each treatment were calculated by pooling the data from 60 cuttings. For greenhouse data, parameters were recorded per cutting, and means were calculated based on each tray. Statistical analysis was conducted using Least Squares Means in JMP Pro 16 (SAS Institute Inc., Cary, NC, USA). If significance occurred, means were separated using Fisher’s LSD test at the *P* ≤ 0.05 level.

### 2.2 Study II – cultivar and rooting hormone trial

#### 2.2.1 Plant materials

A total of 128 tip cuttings were collected from stock plants of ‘MR-Malaysian’, ‘Hawaii’, and ‘DR (Drought-Resistant)-Bumblebee’. The care and maintenance of the ‘MR-Malaysian’ stock plants followed the same practices described in Study I. The ‘Hawaii’ and ‘DR-Bumblebee’ stock plants were originally propagated from clonal cuttings and were grown in 57-L and 19-L containers, respectively, using the same soilless substrate described in Study I. All stock plants of these two cultivars were cultivated in a gutter-connected greenhouse with polycarbonate paneling that reduced light by 30%. Like ‘MR-Malaysian’, the ‘Hawaii’ and ‘DR-Bumblebee’ stock plants were over three years old and had reached reproductive maturity. Fertilization and irrigation practices were consistent with those in Study I, except that rainfall was not supplemented in the greenhouses. Stock plant selection, as well as cutting selection and processing, followed the same procedures outlined in Study I.

#### 2.2.2 Treatment and experiment design

As in Study I, this experiment was conducted in both a greenhouse and an indoor growth room, with setups in both environments consistent with those described in the previous study. In the indoor growth room, each aeroponic unit contained eight cuttings per cultivar, totaling 24 cuttings per unit. The cuttings were randomly arranged within each unit to minimize positional bias and were placed upright using color-coded polyethylene cloning collars (**Figure 2**). The water reservoir of each aeroponic unit was filled with either plain water or a rooting hormone solution containing 5 mg/L IBA and 2.5 mg/L NAA. A total of eight aeroponic units, four with water only and four with hormone solutions, were randomly arranged in the indoor growth room.

In the greenhouse, 64 cuttings from each cultivar were rooted in eight 8-cell trays filled with a soilless substrate consisting of 80% vermiculite and 20% perlite (v:v), with eight cuttings per tray. Half of the trays contained cuttings inserted directly into the substrate, while the other half held cuttings that were quickly dipped in a liquid rooting hormone solution (5 mg/L IBA and 2.5 mg/L NAA) for 5 seconds prior to insertion. The misting schedule was adjusted to prevent leaching during the first 24 hours and to minimize it throughout the experiment to ensure adequate hormone uptake and prolonged hormone retention in the substrate. The trays were randomly arranged on the same mist bench under a shade cloth (70% light exclusion) and were exposed to natural daylight and daylength. As in Study I, six additional sacrifice trays, each containing 18 cuttings with varying combinations of cultivar and hormone treatment, were used for weekly photographic documentation over a six-week period.

The study followed a completely randomized design with four replications per treatment and eight subsamples per replication. For each factor combination, four 8-cell trays served as the four replications, with each cutting within a tray considered a subsample for all measured parameters except rooting percentage. In total, 384 cuttings were analyzed for all measured parameters except rooting percentage. For rooting percentage, four calculated values per treatment were used for analysis.

#### 2.2.3 Environmental conditions and data analysis

Environmental conditions, data collection, and analysis were similar to those in Study I, with the following exceptions: a 14-hour photoperiod was used in the indoor growth rooms, and the number of roots was recorded at the end of the experiment. The environmental conditions are summarized in **Table 1**.

## 3 Results

### 3.1 Study I – photoperiod and seasonal trial

**Figure 3:**
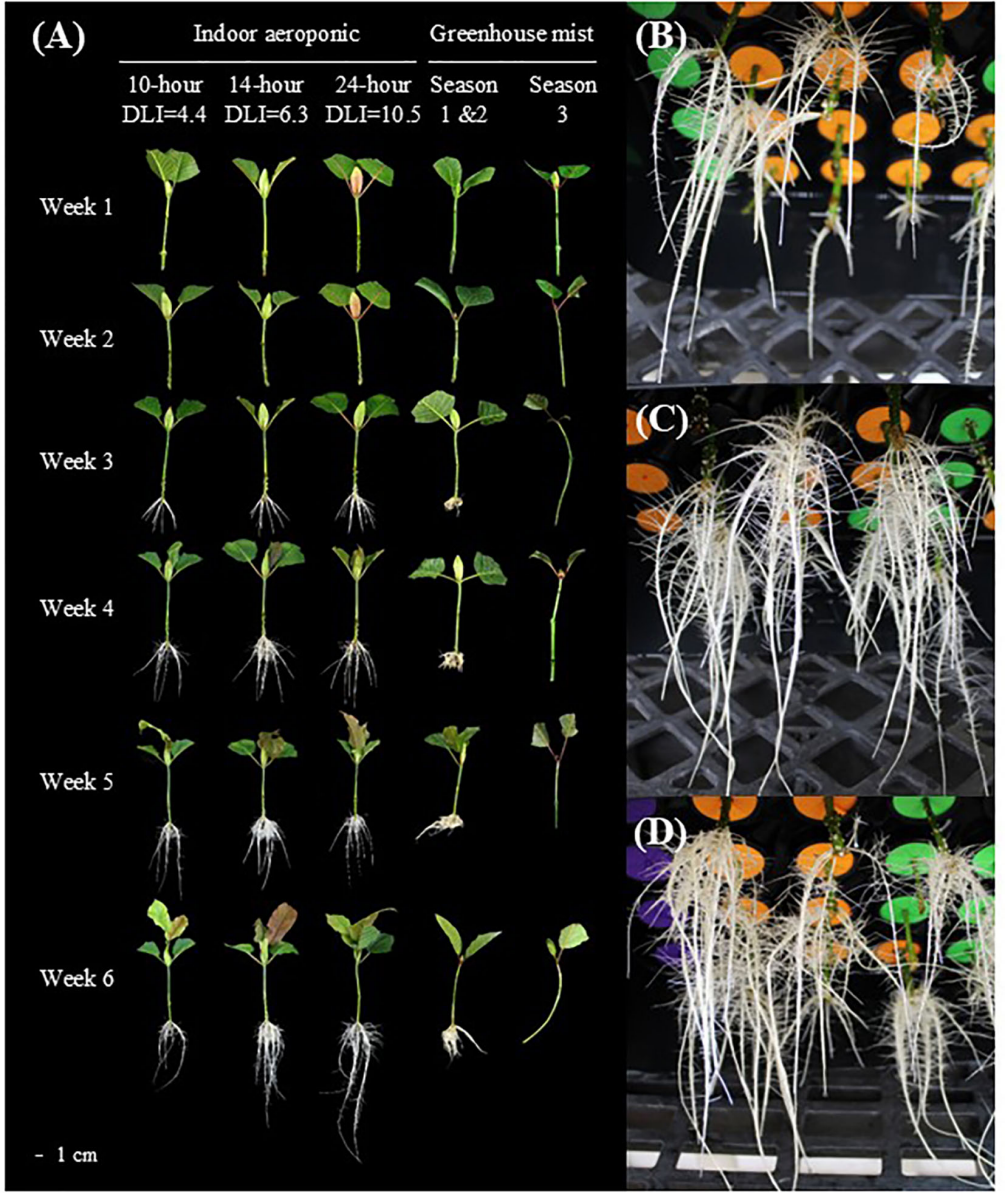
An illustration of the rooting process in indoor aeroponic units and greenhouses. Weekly root development of *Mitragyna speciosa* ‘MR-Malaysian’ cuttings under different lighting treatments, propagation methods, locations, and seasonal conditions in Study I **(A)**. Rooting conditions of *Mitragyna speciosa* ‘MR-Malaysian’ cuttings under a photoperiod of 10 hours **(B)**, 14 hours **(C)**, and 24 hours **(D)** using indoor aeroponic systems at the end of the experiment.

Rooting percentage in the greenhouse varied widely across seasons, ranging from 7% to 98%, while rooting success with indoor aeroponic systems remained relatively stable between 85% and 92% (**Tables 2**, **3**; **Figure 3**). In the greenhouse, little differences were observed between season 1 and season 2 for all measured parameters, including root dry mass, number of new leaves, new leaf area, total root length, root projected area and surface area, average root diameter, and root volume (**Table 2**). However, few cuttings were rooted in season 3, and most of the aforementioned parameters were not recordable. The only exceptions were new leaf number and new leaf area, both of which were zero and significantly lower than in Seasons 1 and 2 (**Table 2**).

**Table 2:** Average (± _S.E._) rooting percentage, root biomass, leaf traits, and root morphological characteristics of greenhouse-propagated *Mitragyna speciosa* ‘MR-Malaysian’ cuttings across three seasons in Study I.

Greenhouse mist	Season 1	Season 2	Season 3
Rooting percentage (%)	98.3 ± 3.3 a	73.3 ± 3.3 b	6.7 ± 3.3 c
Root dry mass (g)	0.072 ± 0.021	0.089 ± 0.010	NA
New leaf number	1.0 ± 0.4 a	1.0 ± 0.2 a	0 b
New leaf area	22.2 ± 9.9 a	22.5 ± 4.3 a	0 b
Total root length (cm)	295.8 ± 88.8	379.8 ± 41.0	NA
Root projected area (cm^2^)	13.1 ± 3.8	14.1 ± 1.4	NA
Root surface area (cm^2^)	41.1 ± 12.0	44.4 ± 4.2	NA
Average root diameter (mm)	0.44 ± 0.01	0.45 ± 0.02	NA
Root volume (cm^3^)	0.45 ± 0.14	0.45 ± 0.03	NA

Data are presented only when more than two valid observations are available. NA, not applicable, fewer than two cuttings rooted per replication. Means followed by different letters are significantly different across seasons by Fisher’s LSD test at P < 0.05.

**Table 3:** Average (± _S.E._) rooting percentage, days to root, root biomass, leaf traits, and root morphological characteristics of *Mitragyna speciosa* ‘MR-Malaysian’ cuttings propagated aeroponically under varying daily light integrals (DLIs) delivered via different photoperiods indoors in Study I.

Indoor aeroponic		10 Hours (DLI = 4.4 mol/m^-2^·d^-1^)	14 Hours (DLI = 6.3 mol/m^-2^·d^-1^)	24 Hours (DLI = 10.5 mol/m^-2^·d^-1^)
Rooting percentage (%)	Season 1	86.7	91.7	86.7
	Season 2	86.7	85.0	90.0
Days to root (d)		22.3 ± 0.5 a	21.1 ± 0.4 ab	19.0 ± 0.9 b
Root dry mass (g)		0.035 ± 0.010 b	0.073 ± 0.008 a	0.060 ± 0.006 ab
New leaf number		1.5 ± 0.2	1.5 ± 0.2	1.4 ± 0.2
New leaf area (cm^2^)		27.04 ± 0.56 ab	42.43 ± 2.97 a	25.22 ± 5.54 b
Total root length (cm)		130.5 ± 12.0 b	250.0 ± 7.3 a	211.9 ± 35.4 ab
Root projected area (cm^2^)		6.51 ± 1.51 b	11.77 ± 0.01 a	10.27 ± 0.90 ab
Root surface area (cm^2^)		20.44 ± 4.73 b	36.97 ± 0.04 a	32.25 ± 2.84 ab
Average root diameter (mm)		0.520 ± 0.025	0.513 ± 0.003	0.544 ± 0.004
Root volume (cm^3^)		0.25 ± 0.07	0.45 ± 0.01	0.37 ± 0.04
Number of root tips		215.5 ± 62.9	370.4 ± 65.5	317.1 ± 26.6
Number of root forks		341.6 ± 17.4	766.9 ± 66.8	741.4 ± 154.4
Number of root crossings		47.5 ± 5.5 b	106.8 ± 0.7 a	95.0 ± 7.3 a

Means within the same row sharing different letters are statistically different by Fisher’s LSD test at P < 0.05.

With indoor aeroponic units, rooting percentages remained stable across photoperiod treatments regardless of season (**Table 3**). However, an increase in photoperiod accelerated root initiation. For example, increasing the photoperiod from 10 to 14 hours promoted root initiation about one day earlier, and further expanding it to 24 hours led to a three-day earlier initiation. Root dry mass was more than doubled when the photoperiod rose from 10 to 14 hours, but an additional increase in photoperiod had minimal impact. The number of new leaves was similar across the three photoperiod treatments, whereas the new leaf area was greatest under the 14-hour photoperiod, being 57-68% larger than under the other treatments.

Overall, root quality improved significantly as the photoperiod increased from 10 to 14 hours, with no further enhancements observed beyond 14 hours (**Table 3**, **Figure 3**). The greatest total root length occurred at the 14-hour photoperiod, which was 92% and 18% greater than the lengths observed at 10- and 24- hour photoperiods, respectively. Similarly, root projected area and surface area increased by 81% from 10 to 14 hours but showed no additional gains at 24 hours. Photoperiod had minimal effects on average root diameter, root volume, and the number of root tips and forks; however, the number of root crossings increased significantly, by 100-125%, under extended photoperiods compared to the 10-hour treatment.

### 3.2 Study II – cultivar and rooting hormone trial

**Figure 4:**
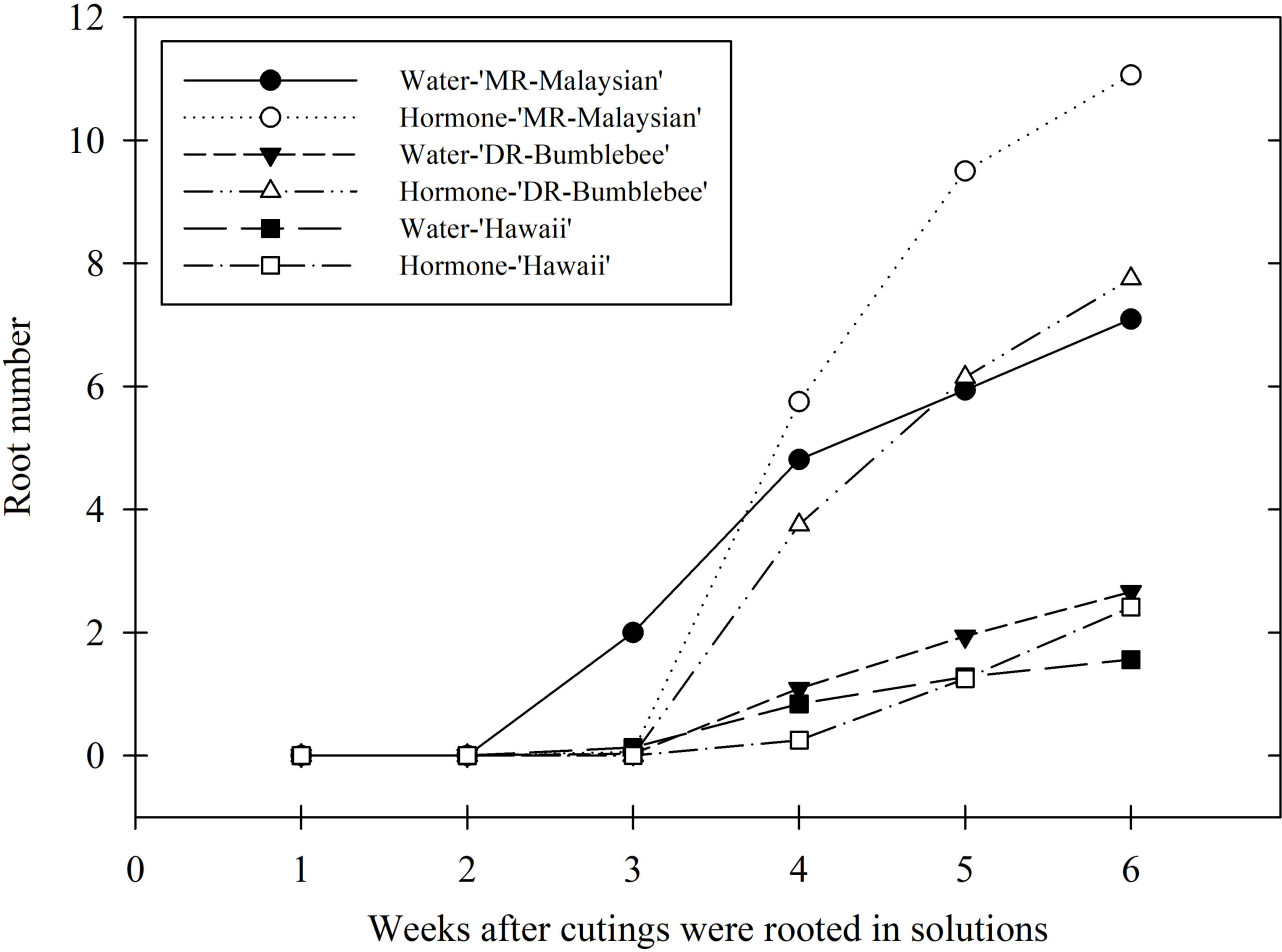
Weekly average root number of three *Mitragyna* *speciosa* cultivars (MR-Malaysian, Hawaii, and DR-Bumblebee), with (hormone-) or without (water-) rooting hormone treatment, in Study II.

Under indoor aeroponic conditions, the MR-Malaysian cultivar achieved the highest rooting success (71-75%), followed by DR-Bumblebee (57-60%), which significantly outperformed Hawaii (~40%) across all hormone treatments (**Table 4**). Root initiation occurred fastest in ‘MR-Malaysian’, averaging 24–27 days, compared to 25–33 days in ‘Hawaii’ and ‘DR-Bumblebee’, although the differences were not statistically significant (**Figure 4**). Rooting hormone application slightly delayed root initiation in ‘MR-Malaysian’ and ‘Hawaii’ (**Figure 4**) but significantly increased the final root number across all three cultivars, particularly in ‘Hawaii’ (**Table 4**), which reduced the disparity between ‘Hawaii’ and the other two cultivars from 144-169% to 42-78% (**Table 4**, **Figure 4**). In the absence of rooting hormone, ‘MR-Malaysian’ and ‘DR-Bumblebee’ produced 144-169% more roots and 113-123% greater root dry mass than ‘Hawaii’. The number of newly formed leaves, regardless of cultivars and hormone treatment, averaged fewer than one per cutting, but ‘Hawaii’ and ‘DR-Bumblebee’ had relatively higher values of new leaf area than ‘MR-Malaysian’. ‘MR-Malaysian’ and ‘DR-Bumblebee’ had comparable values for total root length, root projected area, and surface area, and the number of tips, forks, and crossings (**Table 5**). These values were generally higher than those of ‘Hawaii’, but they were not statistically different. Among the three cultivars, MR-Malaysian had the greatest average root diameter: 37-39% greater than that of DR-Bumblebee and 155-179% greater than that of Hawaii. MR-Malaysian and DR-Bumblebee also exhibited greater root volume, ranging from 62% to 111% higher than that of Hawaii. Rooting hormone application generally enhanced most root traits across all cultivars, though these improvements were not statistically significant. Specifically, rooting hormone increased total root length by 15-27%, projected area and surface area by 20-40%, average root diameter by 6-16%, root volume by 26-44%, number of tips by 15-26%, and number of forks by 12-20%.

**Table 4:** Comparisons of average (± _S.E._) rooting percentage, final root number, root biomass, days to root, and leaf traits of stem cuttings of three *Mitragyna speciosa* cultivars (MR-Malaysian, Hawaii, and DR-Bumblebee) propagated via indoor aeroponics versus greenhouse mist systems, with or without rooting hormone treatments in Study II.

Rooting parameters	Cultivars	Indoor aeroponic		Greenhouse mist	
		Water	Hormone	Water	Hormone
Rooting percentage (%)	MR-Malaysian	71.4 ± 7.1 Aa	74.5 ± 7.1 Aa	38.0 ± 7.1 Ab	31.8 ± 7.1 Ab
	Hawaii	37.8 ± 7.1 Ba	40.9 ± 7.1 Ba	3.1 ± 3.1 Bb	6.3 ± 3.6 Bb
	DR-Bumblebee	56.5 ± 7.1 Aa	59.6 ± 7.1 Aa	23.2 ± 7.1 Ab	16.9 ± 7.1 Ab
Final root number	MR-Malaysian	5.9 ± 0.8 Ab	9.8 ± 0.8 Aa	2.6 ± 0.9 Ac	2.8 ± 0.8 Ac
	Hawaii	1.6 ± 0.8 Bb	5.5 ± 0.8 Ba	0.1 ± 0.1 Bc	0.1 ± 0.1 Bc
	DR-Bumblebee	3.9 ± 0.8 Ab	7.8 ± 0.8 Aa	0.7 ± 0.8 Ac	0.8 ± 0.8 Ac
Root dry mass (mg)	MR-Malaysian	22.3 ± 3.7 Aa	26.6 ± 3.8 Aa	5.4 ± 4.4 Ab	5.5 ± 4.4 Ab
	Hawaii	10.3 ± 3.7 Ba	14.6 ± 3.7 Ba	< 0.1 Bb	< 0.1 Bb
	DR-Bumblebee	21.3 ± 3.7 Aa	25.6 ± 3.9 Aa	4.3 ± 3.8 Ab	4.4 ± 3.8 Ab
Days to root (d)	MR-Malaysian	23.5 ± 2.2	26.6 ± 2.2	NA	NA
	Hawaii	25.3 ± 2.7	32.3 ± 2.7	NA	NA
	DR-Bumblebee	31.4 ± 2.2	28.9 ± 2.2	NA	NA
New leaf number	MR-Malaysian	0.26 ± 0.07 Ba	0.23 ± 0.07 Bab	0.04 ± 0.07 Bb	0.04 ± 0.07 Bb
	Hawaii	0.39 ± 0.07 ABa	0.35 ± 0.07 ABab	0.17 ± 0.07 ABb	0.17 ± 0.07 ABb
	DR-Bumblebee	0.50 ± 0.07 Aa	0.47 ± 0.08 Aab	0.28 ± 0.07 Ab	0.28 ± 0.07 Ab
New leaf area (cm^2^)	MR-Malaysian	0.6 ± 0.5 Bab	1.2 ± 0.5 Ba	0 Bb	0.11 ± 0.11 Bb
	Hawaii	2.1 ± 0.5 Aab	2.7 ± 0.5 Aa	1.0 ± 0.5 Ab	1.0 ± 0.5 Ab
	DR-Bumblebee	2.5 ± 0.5 Aab	3.1 ± 0.5 Aa	1.4 ± 0.5 Ab	1.4 ± 0.5 Ab

Mean comparisons were made separately for each measured trait. Uppercase letters indicate mean separation among cultivars within each column; lowercase letters indicate mean separation for hormone and propagation method treatments within each row. Means sharing the same letter are not statistically different by Tukey’s HSD test at P < 0.05. NA, not applicable.

**Table 5:** Comparisons of average (± _S.E._) root morphological characteristics of stem cuttings of three *Mitragyna speciosa* cultivars (MR-Malaysian, Hawaii, and DR-Bumblebee) propagated via indoor aeroponics versus under greenhouse mist systems, with or without rooting hormone treatments in Study II.

Rooting parameters	Cultivars	Indoor		Greenhouse	
		Water	Hormone	Water	Hormone
Total root length (cm)	MR-Malaysian	69.56 ± 12.41 a	79.85 ± 12.59 a	1.05 ± 0.28 b	1.06 ± 0.55 b
	Hawaii	38.18 ± 12.21 a	48.47 ± 12.45 a	0.02 ± 0.02 b	0.07 ± 0.04 b
	DR-Bumblebee	68.01 ± 12.31 a	78.30 ± 13.19 a	0.11 ± 0.08 b	0.32 ± 0.26 b
Root projected area (cm^2^)	MR-Malaysian	3.959 ± 0.615 a	4.750 ± 0.626 a	0.832 ± 0.615 b	0.807 ± 0.615 b
	Hawaii	2.000 ± 0.615 a	2.790 ± 0.626 a	0.002 ± 0.002 b	0.004 ± 0.002 b
	DR-Bumblebee	3.681 ± 0.619 a	4.471 ± 0.664 a	0.010 ± 0.006 b	0.033 ± 0.029 b
Root surface area (cm^2^)	MR-Malaysian	12.51 ± 1.97 a	15.00 ± 2.00 a	2.84 ± 2.12 b	2.62 ± 1.97 b
	Hawaii	6.24 ± 1.96 a	8.73 ± 2.00 a	0.01 ± 0.01 b	0.01 ± 0.01 b
	DR-Bumblebee	11.52 ± 1.97 a	14.01 ± 2.12 a	0.03 ± 0.02 b	0.10 ± 0.09 b
Average root diameter (mm)	MR-Malaysian	0.53 ± 0.05 Aab	0.56 ± 0.05 Aa	0.37 ± 0.05 Abc	0.31 ± 0.05 Ac
	Hawaii	0.19 ± 0.05 Cab	0.22 ± 0.05 Ca	0.04 ± 0.02 Cbc	0.02 ± 0.02 Cc
	DR-Bumblebee	0.38 ± 0.05 Bab	0.41 ± 0.05 Ba	0.23 ± 0.05 Bbc	0.16 ± 0.05 Bc
Root volume (cm^3^)	MR-Malaysian	0.1909 ± 0.0262 Aa	0.2353 ± 0.0267 Aa	0.0538 ± 0.0281 Ab	0.0501 ± 0.0262 Ab
	Hawaii	0.0856 ± 0.0262 Ba	0.1300 ± 0.0265 Ba	0.0001 ± 0.0001 Bb	0.0003 ± 0.0002 Bb
	DR-Bumblebee	0.1610 ± 0.0262 Aa	0.2055 ± 0.0281 Aa	0.0240 ± 0.0267 Ab	0.0027 ± 0.0025 Ab
Number of root tips	MR-Malaysian	107.1 ± 26.8 a	126.2 ± 26.8 a	2.5 ± 0.4 b	2.0 ± 0.7 b
	Hawaii	72.8 ± 26.4 a	91.9 ± 26.4 a	0.1 ± 0.1 b	0.3 ± 0.2 b
	DR-Bumblebee	131.3 ± 26.4 a	150.4 ± 26.4 a	0.2 ± 0.1 b	1.0 ± 0.6 b
Number of root forks	MR-Malaysian	139.9 ± 35.5 a	159.8 ± 35.9 a	0.8 ± 0.4 b	1.4 ± 0.3 b
	Hawaii	97.6 ± 34.9 a	117.4 ± 35.6 a	0.0 ± 0.0 b	0.0 ± 0.0 b
	DR-Bumblebee	168.7 ± 35.2 a	188.6 ± 37.6 a	0.1 ± 0.1 b	0.3 ± 0.3 b
Number of root crossings	MR-Malaysian	25.4 ± 7.2 a	23.7 ± 7.3 a	0.0 ± 0.0 b	0.0 ± 0.0 b
	Hawaii	17.7 ± 7.1 a	16.0 ± 7.2 a	0.0 ± 0.0 b	0.0 ± 0.0 b
	DR-Bumblebee	34.0 ± 7.2 a	32.4 ± 7.6 a	0.0 ± 0.0 b	0.0 ± 0.0 b

Mean comparisons were made separately for each measured trait. Uppercase letters indicate mean separation among cultivars within each column; lowercase letters indicate mean separation for hormone and propagation method treatments within each row. Means sharing different letters are statistically different by Tukey’s HSD test at P < 0.05.

Similar trends in rooting percentage, final root number, root dry mass, leaf traits, and root morphological characteristics were observed across the three cultivars under mist bench conditions in the greenhouse (**Tables 4**, **5**). ‘MR-Malaysian’ exhibited the highest rooting success (32-38%), followed by ‘DR-Bumblebee’ (17-23%), while ‘Hawaii’ showed significantly lower success (3-6%) (**Table 4**). Although overall root development was limited across all cultivars in the greenhouse, ‘MR-Malaysian’ and ‘DR-Bumblebee’ produced relatively more roots and accumulated greater root dry mass, whereas ‘Hawaii’ exhibited minimal root formation. ‘MR-Malaysian’ consistently showed superior performance in all root morphological traits compared to the other cultivars, but only its average root diameter and root volume differed significantly (**Table 5**). Despite its enhanced root growth, ‘MR-Malaysian’ had the least leaf development, producing the fewest new leaves and the smallest new leaf area relative to ‘DR-Bumblebee’ and ‘Hawaii’ (**Table 4**). Additionally, the application of rooting hormone had a minimal impact on root development in the greenhouse, regardless of cultivar.

Overall, the indoor aeroponic system significantly outperformed the greenhouse mist system across nearly all measured parameters. For example, rooting percentages of ‘MR-Malaysian’ and ‘DR-Bumblebee’ in the aeroponic unit were two-fold greater than the greenhouse, and the differences were more than six-fold for ‘Hawaii’. The final root number and root dry mass also showed substantial gains, increasing by one to four-fold, over 15-fold, and four to eight-fold in MR-Malaysian, Hawaii, and DR-Bumblebee, respectively (**Table 4**; **Figure 4**). Additionally, root morphological traits improved dramatically, with increases exceeding five-fold in MR-Malaysian, 150-fold in DR-Bumblebee, and an exceptional 500-fold in Hawaii (**Table 5**; **Figure 4**).

## 4 Discussion

Propagation of cuttings under a controlled indoor environment holds great potential and offers many benefits for year-round, high-quality transplant production compared to conventional greenhouse mist systems (Gibson et al., 2020). In some woody species, the window for successful rooting is brief, typically occurring from late spring to mid-summer when trees are actively producing new shoots (Cameron et al., 2005). Similarly, in this study, kratom cuttings propagated under a greenhouse mist bench exhibited drastic seasonal variability in rooting success (ranging from 7% to 98%) and inconsistent root growth, sometimes comparable to, but often poorer than, that achieved in indoor aeroponic systems. Under natural conditions, kratom’s active growing period generally spans from April through September, though the use of heat-retentive shade structures can extend this into the winter months. Despite such season-extension techniques, traditional greenhouse mist benches may still fall short in winter due to lower temperatures, reduced humidity, and insufficient photoperiod and DLI. Such factors are particularly limiting for tropical species like kratom. In contrast, indoor aeroponic systems provide a controlled environment that eliminates seasonal fluctuations, consistently delivering high rooting success with better root quality, even for hard-to-root cultivars such as ‘Hawaii’. In this study, root initiation in aeroponic systems occurred reliably between weeks 3 and 5, regardless of cultivar and DLI, with roots fully developed and ready for transplant by week 6 (**Figure 4**). This approach effectively shortens the typical 8- to 10-week propagation cycle observed in greenhouses, even under optimal seasonal conditions. Collectively, our findings support the use of indoor aeroponic systems as a reliable method for year-round production of high-quality kratom cuttings, particularly for challenging cultivars. Greenhouse mist systems, on the other hand, can serve as a cost-effective alternative for propagation during favorable seasonal windows.

Light substantially affects the rooting success of cuttings (Koski et al., 2024), as root initiation, growth, and development depend on an adequate supply of carbohydrates produced through photosynthesis, of which DLI is a crucial factor. DLI can be increased by either raising the instantaneous light intensity or extending the photoperiod. While most propagation studies have focused on increasing DLI through supplemental lighting (Currey et al., 2012; Lopez and Runkle, 2008) or adjusting the photoperiod of stock plants (Koski et al., 2024; Whalley and Cockshull, 1976), relatively few have investigated how photoperiod alone affects rooting success in stem cuttings. Nanda et al. (1967) reported that a short-day photoperiod is necessary for successful root initiation and development in *Bryophyllum tubiflorum* stem cuttings. In the present study, a photoperiod of 14 hours resulted in the most favorable rooting outcomes in kratom cuttings. Because all cuttings were rooted in identical aeroponic units under well-controlled indoor conditions, seasonal effects were minimal. As shown in **Table 3**, cuttings exposed to the 14-hour photoperiod showed the most total root length, projected and surface area, root volume, and number of tips, forks, and crossings. According to Waisel et al. (2002), root projected area represents the root’s appearance on a two-dimensional plane, while surface area refers to the total external area, including root hairs, folds, and irregularities. Both metrics reflect the root’s potential to absorb water and nutrients, which supports plant growth and longevity. Similarly, Cao et al. (2023) described total root length as an indicator of a root system’s development and elongation capacity, with root tips reflecting the physiological metabolic activity. Root forks indicate the breadth of its distribution, while root crossings, encompassing both central and lateral roots, illustrate the root system’s horizontal and vertical distribution area. These traits collectively highlight the critical role of root architecture in supporting metabolic function and resource acquisition, making them strong predictors of transplant survival and long-term plant performance (Larson et al., 2020). Our findings suggest that a 14-hour photoperiod promotes the highest root system functionality in kratom cuttings, indicating enhanced water and nutrient uptake efficiency, as well as improved post-transplant survival. Interestingly, this photoperiod aligns closely with the natural day length in Southeast Asian [typically ranging from 11 to 13 hours year-round (Sengloung et al., 2009; Rengasamy et al., 2022)], the region where kratom is indigenous (Oliveira Neto and Gonçalves, 2019). These results highlight the value of further research to validate the findings and explore how varying light intensities, under a fixed photoperiod, affect kratom rooting, thereby refining lighting guidelines for propagation protocols.

Significant differences in root initiation and root growth were observed among the three kratom cultivars. ‘MR-Malaysian’ consistently demonstrated the highest rooting success and root growth characteristics when rooted in an indoor aeroponic system. Root initiation for ‘MR-Malaysian’ typically occurred within 3 to 4 weeks, depending on seasonal variations, and full root development was generally achieved by week 6. ‘DR-Bumblebee’ exhibited root morphological characteristics comparable to ‘MR-Malaysian’, but with a slightly lower rooting success rate and fewer root numbers overall. Root initiation in ‘DR-Bumblebee’ typically lagged by about one week compared to ‘MR-Malaysian’. Despite this delay, ‘DR-Bumblebee’ was still able to complete root development and reach transplant readiness within the same 6-week cycle. In contrast, ‘Hawaii’ was the most challenging cultivar to root, with rooting success rates typically below 50%. Although root initiation generally occurred between weeks 4 and 5, which is similar to ‘DR-Bumblebee’, the subsequent development was significantly slower. In most cases, ‘Hawaii’ plants were not ready for transplant by the end of week 6, indicating a need for a more extended rooting period or potentially modified propagation conditions to improve outcomes. A similar trend was observed in the greenhouse mist system, although overall rooting performance was lower compared to the indoor aeroponic system.

Plant hormones, primarily auxin, play a crucial role in the propagation of various propagules (Halliday et al., 2009). Since the 1930s, IBA and NAA have been widely used in the horticulture industry for effective rooting of stem cuttings (Sun et al., 2023). Plant responses, however, vary depending on the concentration and formulation of the hormones (Kroin, 2008; Sun et al., 2023). For quick dips, concentrations of 150–500 mg/L are commonly used for herbaceous plants, while 1,000 mg/L is often applied to softwood cuttings (Kroin, 1992). Soaking treatments typically use lower concentrations, ranging from 20–200 ppm for 24-hour exposures (Eigenraam, 2011; Davies et al., 2018). Given that our indoor aeroponic system continuously exposed the cutting base to hormone solution over six weeks, we opted for a lower concentration based on our preliminary, anecdotal observations that higher concentrations, such as 2,500/1,250 mg/L or 7,000/3,500 mg/L IBA/NAA, did not enhance rooting success in kratom and, in some cases, appeared detrimental. Thus, we used 5 mg/L IBA and 2.5 mg/L NAA in this study, which slightly improved kratom rooting percentage and root quality, particularly in aeroponic systems, compared to water control. Although most improvements were not statistically significant, the hormone treatment did significantly increase final root number in the aeroponic system across all three cultivars. This may suggest that the endogenous auxin produced by young kratom shoots and transported to the base of the cuttings is likely sufficient to initiate adventitious root elongation and lateral root emergence. However, exogenous auxin applications appear to support further development. Notably, hormone treatment helped reduce the disparity in rooting performance between easy- and hard-to-root cultivars. For example, in aeroponic systems, the application of rooting hormones reduced the differences between ‘Hawaii’ and the other two cultivars in final root number and root dry mass from 144-169% and 113-123% to 42-78% and 75-82%. This suggests the potential for using rooting hormones to enhance propagation outcomes in challenging genotypes. Further research using a broader range of hormone concentrations is warranted to explore their benefits fully.

Aeroponic propagation offers advantages in reducing the risk of soil-borne diseases. However, it may spread diseases if the solution or propagules carry over pathogens. In this study, *Fusarium* was observed on several kratom cuttings in the indoor aeroponic system. Early symptoms included dark brown necrosis at the cutting tips, often accompanied by a white to translucent gelatinous substance. By Week 4, the percentage of cuttings discarded due to severe *Fusarium* infection was 11% for ‘MR-Malaysian’, 5% for ‘DR-Bumblebee’, and 28% for ‘Hawaii’. Nonetheless, the cultivars still achieved relatively high rooting success rates: 71–75% for ‘MR-Malaysian’, 57–60% for ‘DR-Bumblebee’, and 38–41% for ‘Hawaii’, suggesting that *Fusarium* infection did not universally prevent root development. However, the lower rooting success observed in ‘Hawaii’ may still be partially attributed to *Fusarium* infection. Interestingly, under identical propagation conditions and sanitation protocols (outlined in the Methods section), ‘MR-Malaysian’ appeared to be the least susceptible to *Fusarium*, while ‘DR-Bumblebee’ and ‘Hawaii’ showed greater vulnerability, possibly due to differences in genetic resistance or tissue sensitivity. The infection likely originated from contaminated cuttings, either due to latent infections in stock plants or contamination during handling. This highlights the need for stricter sanitation protocols, particularly in systems prone to pathogen spread, such as aeroponics. In addition to standard sterilization, enhanced measures, such as stock plant disinfection, cutting surface treatments, and targeted fungicide applications, may be crucial in preventing the spread of *Fusarium* and other potential pathogens, thereby ensuring successful propagation.

## 5 Conclusion

To the best of our knowledge, this is the first study to examine the combined effects of multiple factors on kratom cutting propagation across two different cultivation systems. The indoor aeroponic system consistently yielded higher rooting percentages and enhanced root growth throughout the year, whereas the greenhouse mist system demonstrated inconsistent rooting success and variable root growth. A 14-hour photoperiod produced the most favorable rooting performance in kratom cuttings. Among the three kratom cultivars tested, ‘MR-Malaysian’ consistently outperformed the others, showing a high rooting success rate, desirable root morphology, and a relatively short production cycle. ‘DR-Bumblebee’ ranked second in performance, while ‘Hawaii’ was the most challenging cultivar to propagate, with the lowest rooting success rate and the longest production time. The aeroponic system, however, may be susceptible to disease spread if proper sanitation is not maintained. Nevertheless, this study highlights the importance of photoperiod, propagation systems, and cultivar selection in the successful propagation of kratom cuttings. Further research is needed to optimize each of these factors for further improving the efficiency of propagating this valuable medicinal plant.

## Data Availability

The raw data supporting the conclusions of this article will be made available by the authors, without undue reservation.
