# Correction: Aeroponics enables consistent cutting propagation of kratom (*Mitragyna speciosa*): impacts of photoperiod, cultivar, and rooting hormone

**DOI:** 10.3389/fpls.2025.1697035

**Published:** 2025-09-26

**Authors:** Mengzi Zhang, Cynthia Montanez, Brian J. Pearson, Yuncong Li, Jianjun Chen

**Affiliations:** ^1^ Mid-Florida Research and Education Center, Environmental Horticulture Department, University of Florida, Apopka, FL, United States; ^2^ Mid-Columbia Agricultural Research and Extension Center, College of Agricultural Sciences, Oregon State University, Hood River, OR, United States; ^3^ Tropical Research and Education Center, Department of Soil, Water, and Ecosystem Sciences, University of Florida, Homestead, FL, United States

**Keywords:** controlled environment, genotype, hydroponic, medicinal crops, root morphology, root scanning

There was a mistake in **Figure 4**’s X-axis label as published. “Weeks after cuttings were rooted solutions” has been corrected to “Weeks after cuttings were rooted in solutions”. The corrected **Figure 4** appears below.

**Figure 4:**
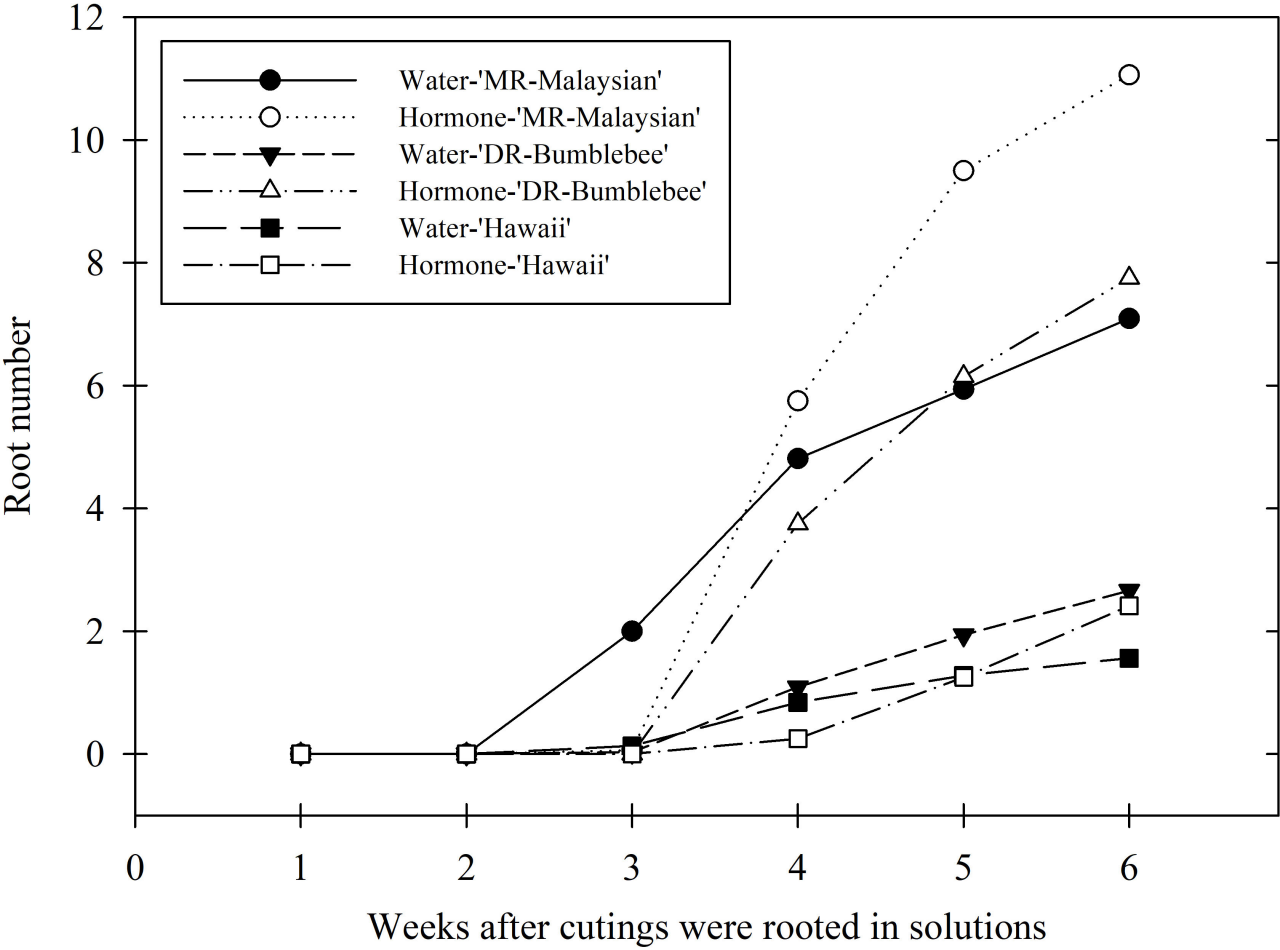
Weekly average root number of three Mitragyna speciosa cultivars (MR-Malaysian, Hawaii, and DR-Bumblebee), with (hormone-) or without (water-) rooting hormone treatment, in Study II.

There was a mistake in **Figure 2**. The drawn image of the aeroponic unit has been updated. The corrected **Figure 2** appears below.

**Figure 2:**
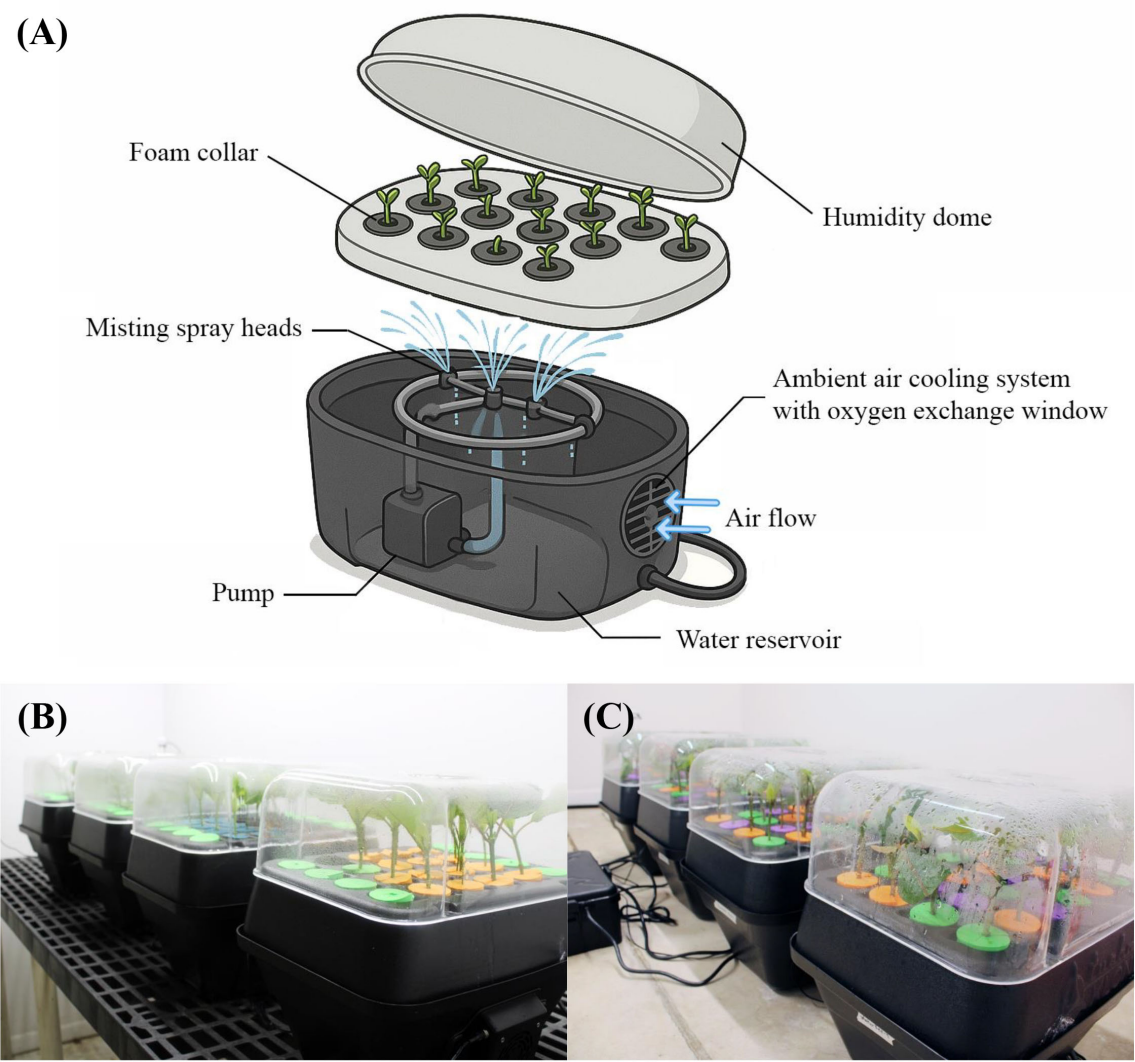
Aeroponic units used in the rooting of Mitragyna speciosa stem cuttings. An illustration of the aeroponic unit **(A)**. The setup of aeroponic units indoors for Study I **(B)** and Study II **(C)** experiments.

The original version of this article has been updated.

